# Exploring Perspectives on a Women in Medicine and Science Mentorship and Networking Event: A Pilot Quality Improvement Initiative at the University of Arizona College of Medicine-Tucson

**DOI:** 10.7759/cureus.108376

**Published:** 2026-05-06

**Authors:** Alexis Hesselbach, Farah Shrourou, Allyson Molzahn, Tejal Parikh

**Affiliations:** 1 Medical School, University of Arizona College of Medicine-Tucson, Tucson, USA; 2 Family and Community Medicine, University of Arizona College of Medicine-Tucson, Tucson, USA

**Keywords:** academic, medicine, mentorship, networking, women

## Abstract

Introduction

Mentorship and networking are critical aspects of career development in academic medicine. Literature suggests that women in medicine have fewer opportunities for mentorship and networking relative to men, including reduced access to mentors and participation in career-advancing social activities. To address this, the Women in Medicine and Science (WIMS) chapter at the University of Arizona College of Medicine-Tucson, Tucson, AZ, USA, hosted a women-centered mentorship and networking event for women medical and premedical students within the university. The purpose of this initiative was to (1) characterize participant perspectives on mentorship and networking for women in medicine, (2) inform the development of additional women-centered mentorship and networking opportunities within the institution, and (3) assess the perceived usefulness of the event as a professional development tool for participants.

Methods

The event was held at the residence of a WIMS faculty mentor to promote a comfortable yet professional space for dialogue. A total of 26 medical and premedical students attended, along with eight women faculty members representing a range of medical specialties. Students were seated in small groups with one faculty member in a round-table setting. Faculty rotated between groups every 20 minutes, ensuring students engaged with multiple physician mentors across specialties. Discussion topics included facing the realities of academic medicine as a woman, balancing personal and professional responsibilities, advice on specialty selection, and perspectives on overcoming barriers to leadership. An anonymous post-event survey was distributed to student participants to assess their views on mentorship and networking for women in medicine, as well as to gauge the usefulness of the event and interest in future events.

Results

Ten out of 26 students completed the survey, yielding a response rate of 38%. Respondents reported that the event provided a transparent, supportive environment for candid discussions with women physicians. Respondents noted that learning about the professional successes of women faculty members was empowering and professionally valuable. Respondents indicated the value of women-centered mentorship and networking opportunities compared to more generalized professional development opportunities. Respondents perceived mentorship and networking for women as supportive of a more inclusive and equitable environment in medicine.

Conclusion

This exploratory, pilot initiative offered insight into the perspectives of medical and premedical students regarding women's professional development within the College of Medicine-Tucson. Despite a modest sample size, single-institution scope, and low survey response rate, feedback from participants supported the value of the mentorship and networking event and the expansion of similar initiatives within the institution. Future projects should refine recruitment approach and methodology to optimize participant response rates, as well as to evaluate long-term outcomes such as longitudinal mentorship, leadership role attainment, specialty selection, and career satisfaction.

## Introduction

Mentorship is an essential component of medical education and training. Previous studies have shown that mentorship is associated with increased career satisfaction, retention, and promotion of faculty members [1]. Despite the predominance of women as medical students and graduates, women are less likely to access strong mentoring relationships compared to men across various stages of training, from residency to faculty member stages [1-2]. This discrepancy is compounded by barriers to networking opportunities, perpetuating gender inequities for women in academic medicine. A 2022 multi-institutional qualitative analysis of academic medical faculty members reported that barriers to networking faced by women include disproportionate personal and familial obligations, as well as exclusion from informal male-dominated activities [3].

At the University of Arizona College of Medicine-Tucson, Tucson, AZ, USA, medical students are involved in various forms of mentorship through participation in educational distinction tracks, research, and specialty-specific interest groups. However, coordinated mentorship and networking opportunities specifically directed toward the professional development of women are less formally represented. While students may seek out these opportunities on an individual basis, the Women In Medicine and Science (WIMS) chapter at the College of Medicine-Tucson sought to provide a structured professional development event focused on women in medicine. The purpose of this event was to provide women medical and premedical students with an approachable yet organized environment to engage with women faculty members. As a pilot quality improvement initiative, participant survey responses were collected to explore perspectives on mentorship and networking for women in medicine, inform the development of future women-centered mentorship and networking opportunities within the institution, and assess the usefulness of the event as a professional development tool for participants. This article was previously presented as an abstract at the 2024 Association of American Medical Colleges (AAMC) Learn Serve Lead Conference in Atlanta, GA, USA, on November 11, 2024.

## Materials and methods

Participants

The University of Arizona College of Medicine-Tucson’s WIMS chapter invited students from the University of Arizona’s medical and undergraduate campuses to attend a mentorship and networking event. Recruitment emails with event descriptions were distributed to one undergraduate premedical interest group and four medical student class cohorts through institutional email lists. Though tailored to the experiences of women in medicine, the event sought to foster an inclusive environment and assess overall student perceptions rather than assessing perceptions by gender. Therefore, invitations were extended to all students regardless of gender identity. A declaration of gender identity was not required for participation. Venue capacity determined the maximum number of potential participants, which was 26. Attendance at the event was voluntary, and participants secured attendance on a first-come, first-served basis through online registration. This initiative was reviewed by the Institutional Review Board (IRB) at the University of Arizona and determined not to constitute human subjects research; therefore, IRB review and approval were not required. 

Networking event

The event was held at the home of a WIMS faculty member. The event was 2.5 hours in duration. Eight women physicians of varying specialties were in attendance. Speciality representation was based on faculty availability. A total of five to eight students were seated at a table with a physician faculty member, with physicians rotating between tables every 20 minutes. Conversations were open-ended and student-directed, covering topics including experiences of women in academic medicine, specialty-specific considerations, and general career advice. At the conclusion of the event, students were given additional time to freely network with faculty members.

Data collection

A single post-event survey was developed by the authors to explore the views of medical and premedical student participants across the following domains: usefulness of the event and setting; exposure to physicians of diverse medical specialties; discussion of gender-related obstacles in medicine; and distinctions between women-centered and generalized mentorship and networking events, including perspectives on diversity, equity, and inclusion. A pre-event assessment was not conducted. As the survey instrument was designed to capture participant perceptions and provide event-specific feedback, formal validation and pilot testing were not conducted. The full survey instrument is listed in Appendix A.

The survey included 20 questions in the format of a 5-point Likert scale (1= strongly disagree, 5 = strongly agree). The survey was concluded with an optional open-ended question inviting participants to provide additional feedback. This survey was distributed to attendees via email after the event. Participation was voluntary, and no incentives were offered for completion of the survey. Participants were informed of the anonymity of their responses. 

Data analysis

Survey responses were compiled in Google Sheets (Google LLC, Mountain View, CA, USA). Descriptive statistics were generated. All 20 Likert-scale items required a response prior to survey submission; therefore, there were no missing quantitative responses. For each Likert-scale item, the proportion of respondents selecting each of the five options was calculated and reported. No inferential statistical analysis was conducted. The final open-ended question was optional, and narrative responses were reviewed for common themes, which were descriptively summarized.

Visualizations were created in Python (version 3.9.6; Python Software Foundation, Wilmington, DE, USA) with Matplotlib (Matplotlib Development Team) and NumPy (NumPy Developers).

## Results

Participant demographics

Of the 26 students who attended the event, 10 students completed the survey. There were eight physicians from various specialties (Table 1).

**Table 1:** Participant demographics among students and physicians

Participant demographics	n
Students	
Medical students	18
Undergraduate students	8
Survey respondents	10
Physician attendees by specialty	
Surgery	1
Family Medicine	2
Internal Medicine	1
Emergency Medicine	2
Obstetrics and Gynecology	1
Diagnostic Radiology	1

Event usefulness

**Figure 1:**
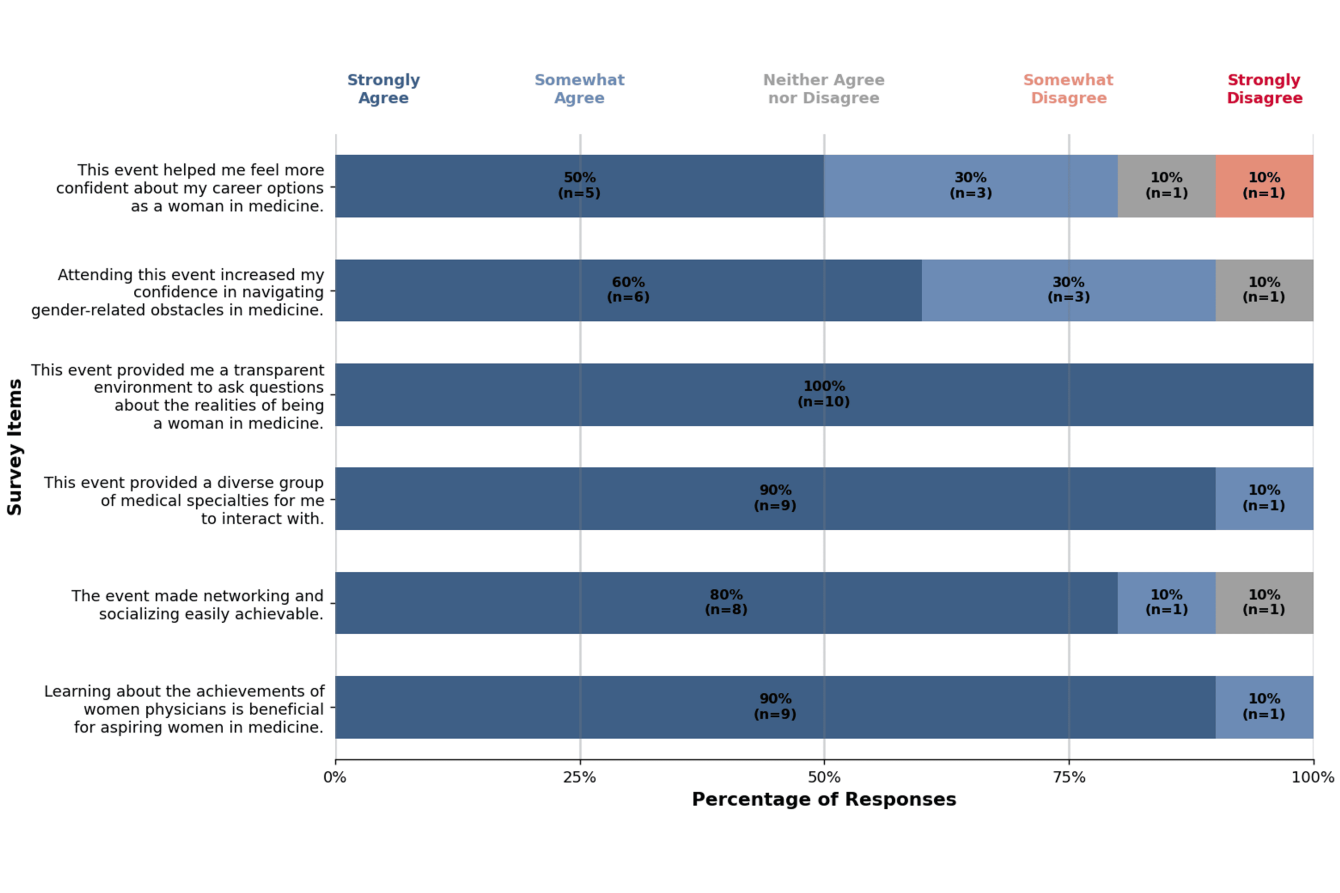
Likert-scale representation of event usefulness

There were six statements addressing the perceived usefulness of the event pertaining to specialty exploration and career options (Figure 1).

Mentorship and networking

**Figure 2:**
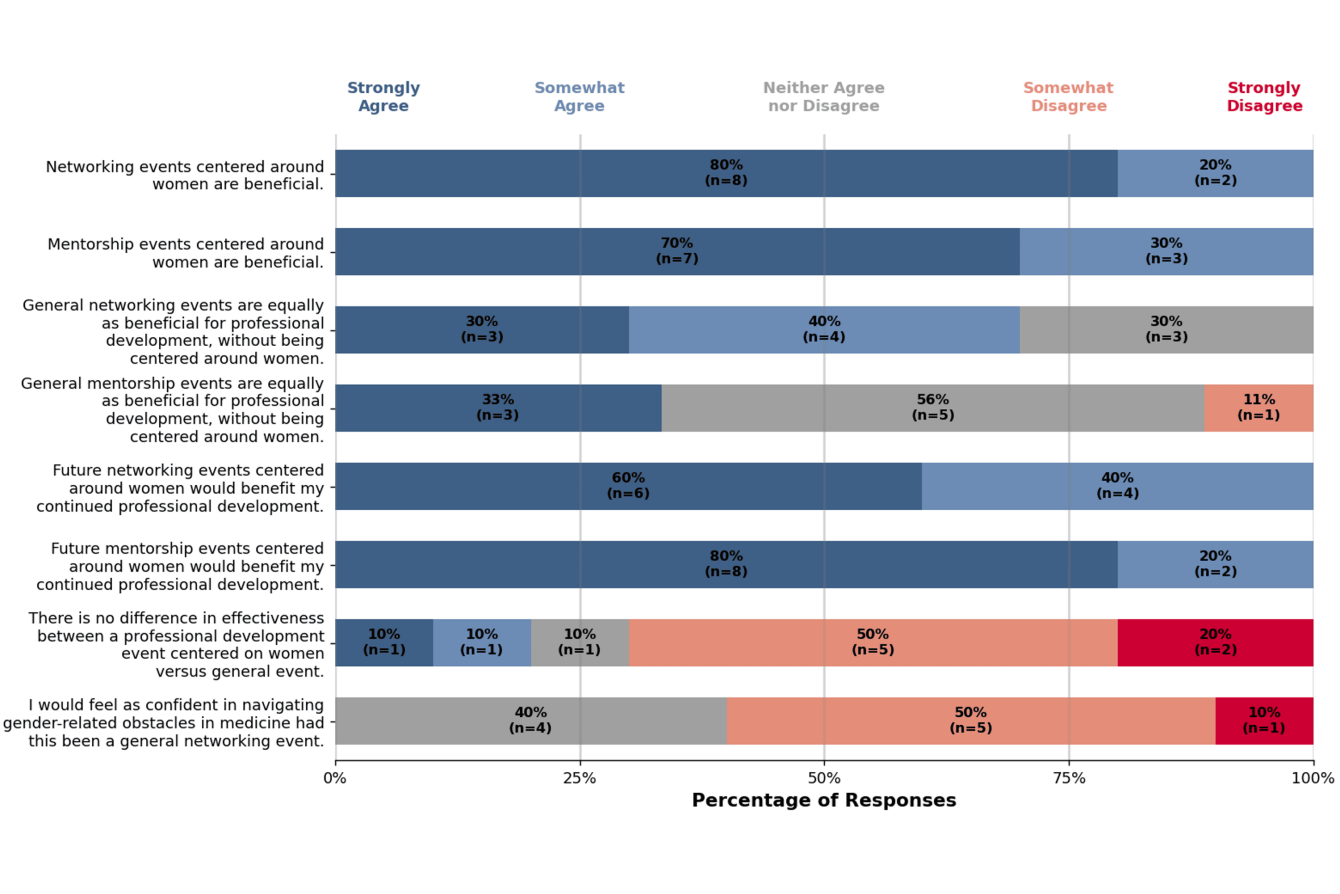
Likert-scale representation of mentorship and networking outcomes of the event

There were eight statements addressing the importance of women-centered mentorship and networking (Figure 2).

Diversity, equity, and inclusion

**Figure 3:**
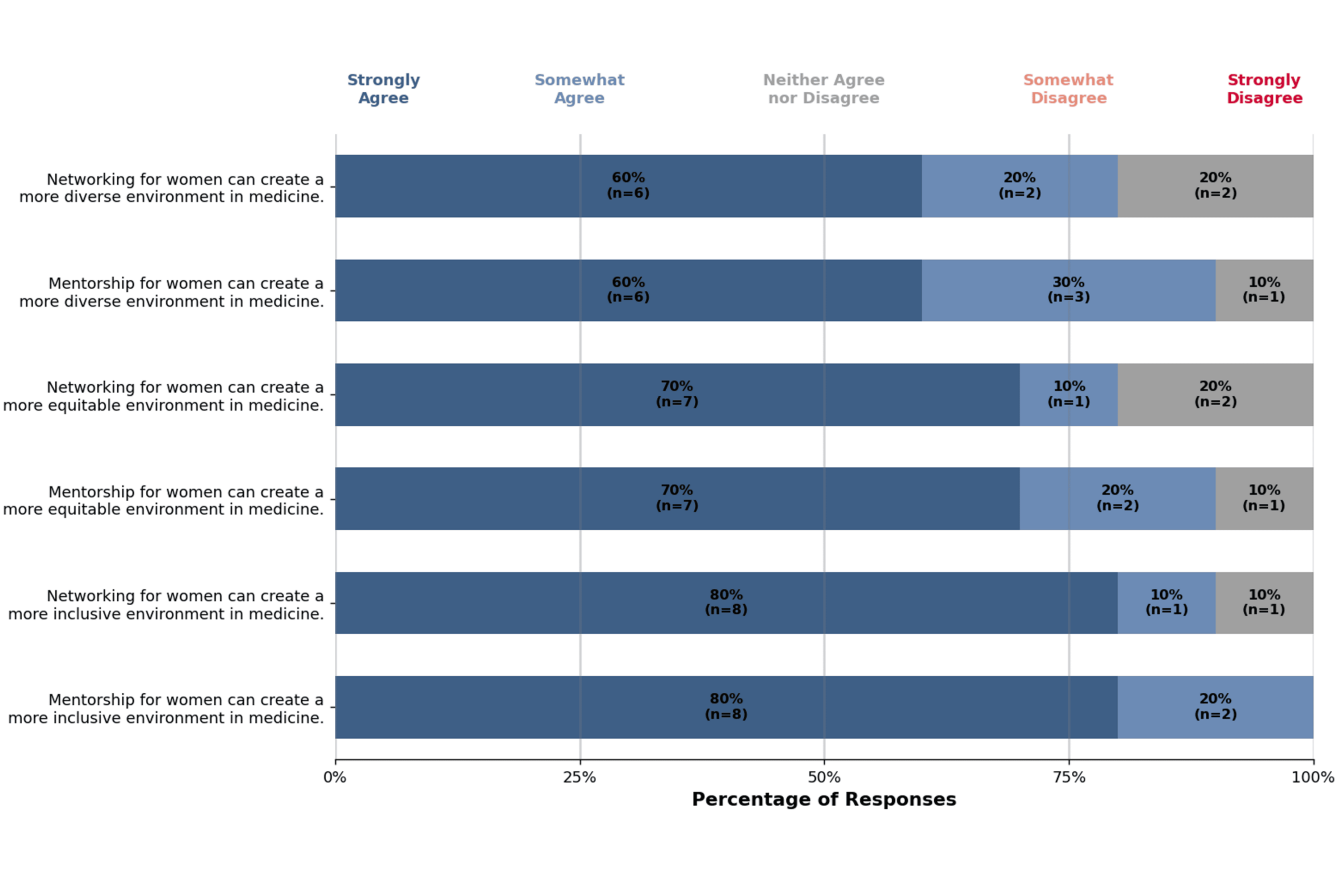
Likert-scale representation of diversity- and inclusion-related outcomes of the event

There were six statements addressing perspectives on diversity, equity, and inclusion in medicine with respect to mentorship and networking (Figure 3).

Open-ended responses

Responses to the optional open-ended survey question were reviewed descriptively by the authors, and recurring themes were summarized. Commonly identified themes included general enthusiasm towards the event overall, with respondents noting the setting was comfortable and conducive to candid, productive conversation. Additionally, respondents shared that the opportunity to engage with successful women physicians and fellow students in this setting was inspiring and professionally valuable.

## Discussion

The findings of this pilot quality improvement initiative reflect previously identified themes within the literature, particularly with respect to women’s professional development within medicine. Prior research has suggested that women-focused professional events facilitate career advancement through exposure to leadership experiences, mentorship, and sponsorship opportunities [4]. Despite the recognized value of mentorship and networking opportunities by women in medicine, there is a relative shortage of such gender-specific programming within academic medical institutions [3]. Here, respondents expressed interest in future women-centered mentorship and networking events and reported greater confidence in navigating gender-related obstacles in medicine. This finding is particularly relevant given the well-documented disparities in career satisfaction, institutional promotion, and research output reported by women in medicine compared to their male counterparts [5-6]. Among traditionally male-dominated fields such as surgery, this phenomenon is especially prevalent [7].

Ongoing gender inequity in academic medicine may be influenced by women's reduced participation in informal networking events, which have been shown to confer significant professional advantages. This lack of participation is multifactorial in etiology. In one study, major barriers for women included a lack of awareness of such events and conflicting caregiving responsibilities, limiting their attendance [8]. In addition, informal networking events historically tailored to men’s sporting and social outings have resulted in the intentional and unintentional exclusion of women [3]. Respondents perceived increased mentorship and networking opportunities for women as supportive of equity and inclusivity within medicine. While women are increasing their representation within medicine, many continue to encounter systemic challenges to professional advancement, including disparities in compensation and matriculation into high-paying specialties, gender-based discrimination, and other persistent implicit biases [9]. These factors are amplified for women of color and those who identify with other underrepresented ethnic, religious, and cultural groups [9]. 

Strengths of this quality improvement initiative include the setting of the event, which provided students with an approachable, round-table format to engage in conversation with potential women faculty mentors. To our knowledge, this represents the most recent initiative conducted at the University of Arizona College of Medicine-Tucson that assesses the perspectives of women premedical and medical students on mentorship and networking with women attending physicians.

There are several methodological limitations that should be considered while interpreting the results. Primary limitations include the small sample size, lack of baseline measures, modest survey response rate, absence of a validated survey instrument, and single-institution focus. Overall, these factors weaken the generalizability of our findings. As participants self-selected for this event, selection bias is likely reflected in respondents’ perceptions of the event’s usefulness. A response rate of 38% and a small sample size inherently limit the extent of conclusions that can be drawn and do not capture the perspectives of all the event participants. Additionally, the delayed distribution of the post-event survey may have introduced recall bias into the results and contributed to lower response rates. Low response rates may be further explained by competing clinical and academic obligations of the student participants. Nonresponse bias is also a concern, as participants with stronger opinions on the event may have been more likely to complete the survey. Furthermore, a validated survey instrument was not implemented, which impacts the interpretability of the results. The single-institution scope of this initiative reduces the generalizability and replicability of our findings beyond this setting. Additionally, our findings reflect only short-term perspectives from a small, self-selected cohort of students, as long-term outcomes were not assessed. 

The findings of this initiative have informed the expansion of WIMS mentorship and networking events for future classes within the College of Medicine-Tucson. Future research regarding similar WIMS initiatives should address the methodological limitations of this project, including improved recruitment and data collection strategies to increase participation and to comprehensively reflect student perspectives. Research on women-centered professional development has largely been subjective and qualitative, with inconsistent evidence on tangible outcomes such as promotion and retention [10]. In the future, we hope to address this by including objective outcomes to better evaluate the impact on participants. Longitudinal studies tracking student and mentor relationships may also provide valuable insight into how early mentorship and networking experiences can influence career advancement, confidence in navigating gender-related challenges, and retention of women in academic medicine. Future investigations might also examine the impact of women-centered versus general networking events across different career stages and how institutional policies and support structures can optimize mentorship opportunities for women. By assessing the evolving professional needs and perspectives of women premedical students, medical students, and physicians, such efforts can contribute to a more equitable and inclusive atmosphere in medicine.

## Conclusions

The findings of this exploratory, pilot quality improvement initiative indicate that participants found the event professionally valuable and perceived women-centered mentorship and networking events as beneficial to their professional development. Additionally, participants reported that similar programming may function as a part of larger institutional efforts related to equity and inclusivity within medicine. However, these findings are based on the short-term perspectives of a small, self-selected cohort of students, which limits generalizability. Despite this limitation, these perspectives are broadly aligned with prior literature highlighting the importance of mentorship and networking for women in medicine. Moreover, these perspectives have informed the expansion of similar WIMS professional development opportunities within the College of Medicine-Tucson. Future multi-institutional studies are needed to build upon these findings with greater methodological rigor, including the use of validated survey instruments, objective outcome measures, and longitudinal follow-up to improve professional development for women in academic medicine. 

## Appendices

Appendix A

Post-event Survey Instrument

**Table 2:** Post-event survey instrument Response scale: 1 = Strongly Disagree, 2 = Disagree, 3 = Neither Agree nor Disagree, 4 = Agree, 5 = Strongly Agree

Question #	Item	Strongly Disagree	Disagree	Neither Agree nor Disagree	Agree	Strongly Agree
Q1	This event helped me feel more confidentabout my career options as a woman in medicine.					
Q2	Attending this event increased my confidence in navigating gender-related obstacles in medicine.					
Q3	This event provided me a transparent environment to ask questions about the realities of being a woman in medicine.					
Q4	This event provided a diverse group of medical specialties for me to interact with.					
Q5	This event made networking and socializing easily accessible and achievable.					
Q6	Mentorship events centered around women are beneficial.					
Q7	Networking events centered around women are beneficial.					
Q8	General networking events are equally as beneficial for professional development, without being centered around women.					
Q9	General mentorship events are equally as beneficial for professional development, without being centered around women.					
Q10	Future networking events centered around women would benefit my continued professional development.					
Q11	Future mentorship events centered around women would benefit my continued professional development.					
Q12	There is no difference in effectiveness between a professional development event centered on women versus a general event.					
Q13	I would feel as confident in navigating gender-related obstacles in medicine had this been a general networking event.					
Q14	Mentorship for women can create a more diverse environment in medicine.					
Q15	Networking for women can create a more diverse environment in medicine.					
Q16	Mentorship for women can create a more equitable environment in medicine.					
Q17	Networking for women can create a more equitable environment in medicine.					
Q18	Mentorship for women can create a more inclusive environment in medicine.					
Q19	Networking for women can create a more inclusive environment in medicine.					
Q20	Learning about the achievements of women physicians is beneficial for aspiring women in medicine.					
Free Response	Optional free response feedback (open-ended):					
